# A New Crown

**Published:** 1884-12

**Authors:** 


### A New Crown.

Within the last few days I have had an opportunity to examine a new method of attaching artificial crowns to roots of teeth, devised by Dr. C. S. Case, of Jackson, Mich. The method seems altogether practicable, and in some respects better than any method heretofore suggested. The adaptation and attachment is more practicable and will be found easier of accomplishment than most of the methods recently presented. Teeth and materials for it are being prepared, and will ere long be avail- to the profession. We hope, by the next issue of the REGISTER, to present a description of the process, with illustrations. This method is one which, when seen, will produce conviction as to its efficiency and value. We only make this mention of the method to prepare for its full description.

